# Diversity and specificity of microsatellites within *Aspergillus* section *Fumigati*

**DOI:** 10.1186/1471-2180-12-154

**Published:** 2012-07-28

**Authors:** Ricardo Araujo, António Amorim, Leonor Gusmão

**Affiliations:** 1 IPATIMUP, Institute of Molecular Pathology and Immunology, University of Porto, Rua Dr. Roberto Frias, s/n, 4200-465, Porto, Portugal; 2 Faculty of Sciences, University of Porto, Rua do Campo Alegre s/n, 4169-007, Porto, Portugal

**Keywords:** *Aspergillus fumigatus*, Invasive aspergillosis, Molecular identification, Multiplex PCR, Short tandem repeats, Microsatellites

## Abstract

**Background:**

Microsatellites (or short tandem repeats, STRs) are the genetic markers of choice for studying *Aspergillus fumigatus* molecular epidemiology due to its reproducibility and high discrimination power. However, the specificity of these markers must be investigated in a group of isolates from closely related species. The aim of this work was to test a microsatellite-based PCR multiplex previously designed for *A. fumigatus* in a set of species belonging to section *Fumigati*, namely *Aspergillus fumigatiaffinis*, *Aspergillus lentulus*, *Aspergillus novofumigatus*, *Aspergillus unilateralis*, *Aspergillus viridinutans, Neosartorya fischeri*, *Neosartorya hiratsukae*, *Neosartorya pseudofischeri* and *Neosartorya udagawae.*

**Results:**

The reference *A. fumigatus* strain ATCC 46645 was easily genotyped in standard conditions showing a final electrophoretic profile of 8 expected peaks corresponding to each microsatellite locus. Inversely, no peaks were observed for all other species from section *Fumigati*, with an exception for marker MC6b in *A. unilateralis*. By screening the genome sequence of *Neosartorya fischeri* NRRL 181, the results showed that MC3, MC6a and MC7 might be employed for *N. fischeri* genotyping since these markers present several repeats of each motif. The accumulation of insertions and deletions was frequently observed in the genomic regions surrounding the microsatellites, including those where the *A. fumigatus* primers are located. The amplification of microsatellite markers in less stringent amplification conditions resulted in a distinct electrophoretic profile for species within section *Fumigati*.

**Conclusions:**

Therefore, the microsatellite-based PCR multiplex allow simple identification of *A. fumigatus* and, with a slight modification of temperature conditions, it also allows discriminating other pathogenic species within section *Fumigati*, particularly *A. fumigatiaffinis, N. fischeri* and *N. udagawae*.

## Background

Molecular diagnosis of fungal diseases has become increasingly more used in clinical laboratories and new species morphologically similar to *Aspergillus fumigatus* were surprisingly revealed [1,2]. Section *Fumigati* includes fungal species closely related to *A. fumigatus* that can go from the anamorphous *Aspergillus* species to the teleomorphic species of the genus *Neosartorya*[3]. Misidentification of fungal species within section *Fumigati* was sporadically reported in some laboratories, particularly of fungal isolates afterwards identified as *Aspergillus lentulus, Aspergillus viridinutans, Aspergillus fumigatiaffinis, Aspergillus fumisynnematus, Neosartorya pseudofischeri, Neosartorya hiratsukae* and *Neosartorya udagawae*[1,2,4,5]. These species present similar microscopical and macroscopical features to *A. fumigatus* and, therefore, molecular identification is at present recommended for the correct identification of species within section *Fumigati*. A set of genes, namely actin, calmodulin, internal transcribed spacer (ITS), rodlet A and/or β-tubulin, has been proposed for a correct identification of *A. fumigatus* and related species following sequencing analysis [3,6]. Multilocus sequence typing (MLST) [4], random amplified polymorphic DNA [7], restriction fragment length polymorphism [8] and microsphere-based Luminex assay [9] may allow molecular identification of *A. fumigatus*. Recently, a practical and cheap electrophoretic strategy was described for molecular identification of *A. fumigatus* and distinction of the species within the section *Fumigati*[10].

In addition to fungal identification, genotyping methodologies have been improving and gained importance in clinical laboratories. Microsatellite typing methods are very useful for studying *A. fumigatus* molecular epidemiology due to its reproducibility and high discrimination power (around 0.9997). A group of eight microsatellite markers combined in a single PCR multiplex assay with high discrimination power is currently available for *A. fumigatus* genotyping [11]. Such tool may be very useful to investigate outbreaks in clinical units, to evaluate quality control programmes particularly in units admitting critical-care patients, to identify patients with chronic fungal colonization (e.g. some cystic fibrosis patients) and patients with invasive disease caused by multiple fungal strains [11-14]. In addition, genotyping approaches might allow evaluating the response of patients to the antifungal therapies [12].

Few microsatellites (or short tandem repeats - STRs) have been described as species-specific [15-18], while others are transversal to a group of closely related species [19]. Nevertheless, these markers are of extreme utility for population and conservation genetics. The complete genome sequence of *Neosartorya fischeri*, a sibling species, was recently published and high homology was revealed when comparing to *A. fumigatus*. Repeat elements density was very similar when comparing these two species and two strains of *A. fumigatus*[20]. The genomic dynamics for acquisition and removal of microsatellites in closely related species is still unknown, and therefore, it is of scientific relevance to compare and highlight the diversity of some microsatellites in a group of very closely related fungi.

*Aspergillus fumigatus* is one of the most common agents of systemic mold infections. Genotyping strategies (mostly employing microsatellites) have been described as very useful in labs for detection of outbreaks, identification of patients chronically colonized with *A. fumigatus* and monitoring of antifungal efficacy in patients [2,5]. In addition, sibling species within section *Fumigati* should also be promptly identified as they present considerable differences in antifungal resistance [21]. The specificity of microsatellite-based PCR multiplex to *A. fumigatus* was first confirmed in a group of *Aspergillus* species [11], but it is also important to assess both the specificity and the diversity of these microsatellites within *Aspergillus* section *Fumigati*. Therefore, the two aims of this study were to evaluate the specificity of a set of previously described microsatellite markers to *A. fumigatus*[11] in a group of closely-related species and the ability of the multiplex to identify *A. fumigatus* and other species belonging to section *Fumigati* based on the presence/absence of some microsatellite markers.

## Results

### Standard microsatellite-based multiplex PCR tested with *Aspergillus* spp. and *Neosartorya* spp

**Figure 1:**
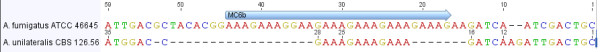
**Alignment of the marker MC6b sequences in *Aspergillus fumigatus* and *Aspergillus unilateralis* .**

A set of eight microsatellites previously described for *A. fumigatus* genotyping strategy was tested with other species belonging to *Aspergillus* section *Fumigati*, namely *A. fumigatiaffinisA. lentulusA. novofumigatusA. unilateralisA. viridinutans, N. fischeri, N. hiratsukaeN. pseudofischeri*, and *N. udagawae*, and a reference strain of *A. fumigatus*. The reference *A. fumigatus* ATCC 46645 was easily genotyped with the standard multiplex conditions and a profile of eight peaks was produced after electrophoretic separation, each one corresponding to a single microsatellite (see Additional file Figure A 1). Similar profile was observed for the remaining ten isolates of *A. fumigatus*, as previously described [11,12]. A similar approach was followed for non-*fumigatus* fungal isolates. No specific PCR amplification products were observed for all tested species from section *Fumigati*, with the exception of MC6b in *A. unilateralis*. Sequence analysis of MC6b in *A. unilateralis* confirmed that this genomic sequence was similar to the sequence of *A. fumigatus* (Figure 1), therefore excluding unspecific amplification of other genomic regions. Nevertheless, the multiplex conditions previously described for *A. fumigatus* genotyping proved to be highly specific, even with the amplification of MC6b in *A. unilateralis*, as the set of eight microsatellite markers could be uniquely observed in *A. fumigatus* isolates.

### Microsatellites in *A. fumigatus* AF293 *versus N. fischeri* NRRL 181

**Figure 2:**
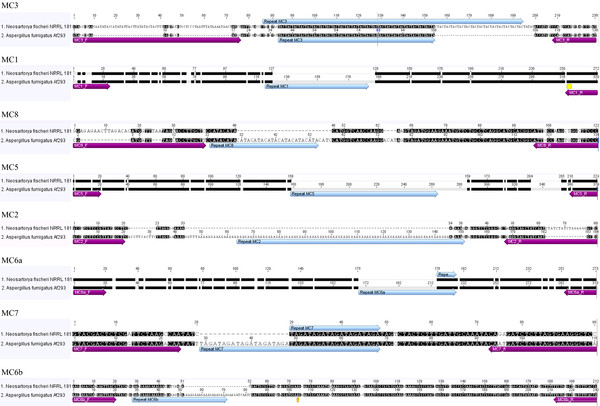
**Alignment of eight microsatellites sequences in *Neosartorya fischeri * NRRL 181and *Aspergillus fumigatus * AF293 (similar nucleotides in both sequences are marked black while polymorphic sequences are marked white).** The primers proposed for microsatellite amplification are marked, as well as the microsatellites within the sequence.

We screened the complete genome sequence of *N. fischeri* NRRL 181 in order to locate and compare the microsatellite markers employed for *A. fumigatus* genotyping. Few microsatellites previously described in *A. fumigatus* were also found in *N. fischeri* genome, with a single one having more than 30 repetitive motifs (e.g. MC3), while other genomic regions were found more stable without the ability to accumulate repeats. Markers MC3, MC6a and MC7 showed sequences with more than three repeats of the original motif detected in *A. fumigatus*, representing microsatellites that are potentially polymorphic and might be employed for *N. fischeri* genotyping. Figure 2 shows a set of eight genomic sequences in *N. fischeri* previously described to be unstable in *A. fumigatus*, representing microsatellites. Curiously, the accumulation of insertions and deletions in these genomic regions was frequently observed, including the regions where the *A. fumigatus* primers were located. Thus, some markers are not expected to be amplified in *N. fischeri* due to extensive modifications of primer regions in the genome of this fungus, as it is the case of MC3, MC1 and MC8 forward primers and MC2 reverse primer (Figure 2).

### Microsatellite-based PCR multiplex for identification of fungal species

We have confirmed the specificity of the microsatellite multiplex for *A. fumigatus* within section *Fumigati* with a single exception observed in *A. unilateralis* (marker MC6b). However, it could not be discarded the detection of few other markers in species belonging to section *Fumigati* if less stringent PCR conditions were employed, as some markers were found in the genome of *N. fischeri* NRRL 181. Therefore, we had tested distinct amplification temperatures (from 48 to 60°C) in the group of species belonging to section *Fumigati*. Few markers could be amplified after decreasing the PCR annealing temperature from 60°C to 55°C (see Table 1). Eight peaks previously observed in *A. fumigatus* were similarly found when testing less stringent PCR conditions. Sequencing analysis of those amplicons revealed genomic similarities to *A. fumigatus* (see Additional file Table A 1; a single exception was MC3 primers that amplified an unspecific region). Remarkably, distinct electrophoretic profiles were obtained for all tested species based on the amplification of the microsatellite multiplex panel at 55°C, as seen in Table 1. The relevant pathogens of section *Fumigati*, *A. fumigatiaffinis, N. fischeri* and *N. udagawae*, were clearly distinguished from *A. fumigatus* and from all the other species within this section. In addition, *A. novofumigatus* was also identified. Besides *A. fumigatus* isolate, MC6a was uniquely amplified with *N. fischeri* isolate, while MC8 was obtained exclusively with *N. udagawae*. The marker MC5 was amplified with *A. fumigatiaffinis* and *A. novofumigatus* (Table 1). Few microsatellites showed more than three repeat motifs, as it was the case of MC6a in *A. lentulus* and MC6b in *A. unilateralis* (sequence analysis of the amplified markers was added as supplementary Table A 1). Sequence analysis of marker MC6b showed that *A. lentulus* and *A. viridinutans* (the most relevant species in clinics besides *A. fumigatus*) were different from all the other tested species.

**Table 1:** **List of markers amplified at 55°C annealing temperature in the group of species belonging to section *Fumigati* **

	**MC3**	**MC1**	**MC8**	**MC5**	**MC2**	**MC6a**	**MC7**	**MC6b**
*Aspergillus fumigatus* ATCC 46645	√	√	√	√	√	√	√	√
*Aspergillus fumigatiaffinis* CBS 117186	√ ^a^			√				√
*Aspergillus lentulus* CBS 116880^b^	√ ^a^							√
*Aspergillus novofumigatus* CBS 117519	√ ^a^			√				
*Aspergillus unilateralis* CBS 126.56	√ ^a^							√
*Aspergillus viridinutans* CBS 121595	√ ^a^							√
*Neosartoryafischeri* CBS 316.89	√ ^a^			√		√		√
*Neosartoryahiratsukae* CBS 124073	√ ^a^							√
*Neosartoryapseudofischeri* CBS 208.92^b^	√ ^a^							√
*Neosartoryaudagawae* CBS 114217	√ ^a^		√					√

a) Unspecific amplification with MC3 primers (confirmed after sequence analysis).

b) Similar results were observed with other tested reference strains.

## Discussion

Species such as *A. lentulus, A. viridinutansN. pseudofischeri*, and *N. udagawae* have been described as human pathogens associated to severe cases of trabecular bone invasion, cutaneous, cerebral, liver or pulmonary aspergillosis [1,2,21-23]. In addition, some species were reported as primary resistant *in vitro* to the substance class of azole antifungals [6,24]. Therefore, due to their intrinsic resistance, infections caused by strains of these species cause difficult to treat infections that deserve increased attention by clinicians. Molecular techniques are recommended for the correct identification of species within the group “*A. fumigatus* complex”, but most clinical laboratories still cannot afford to routinely implement sequencing technologies. Few electrophoretic methodologies are available for molecular identification of *A. fumigatus* and related species and represent valid alternatives [7-10]. Since genotyping strategies have been strongly recommended by researchers, clinicians and technicians to be implemented in clinical laboratories, it would be desirable to combine both identification and genotyping capabilities in a single method. In this study, we explored the specificity of an *A. fumigatus* microsatellite genotyping panel in a group of closely related fungal species. The specificity of microsatellite multiplex was confirmed similar to previously described for other standard molecular methodology, such as MLST [4]. In fact, *A. fumigatus* could be correctly identified employing this strategy, similarly to what was previously described for *Candida parapsilosis*[18], *Cryptococcus neoformans*[15], *Paracoccidioides brasiliensis*[17], and *Saccharomyces boulardii*[16] when using microsatellite markers combined in a multiplex. It is worth mentioning that simplified methodologies based on restricted genotyping panels of only one or two microsatellite markers [e.g. [25], although more practical and rapid for epidemiological studies, can produce inaccurate results. Our data adds to the increasingly reported application of microsatellite alleles to identify some fungi within complexes of species. In this study we also noticed a low transferability of microsatellites within section *Fumigati*, namely when comparing *N. fischeri* genome. A small number of markers (4 of 25) have also been described as transferable from related Uredinales species to *Hemileia vastatrix*[26]. Our results of section *Fumigati* agree with previous reports that describe a smaller fraction of cross species transfer of microsatellites within fungal genera when compared with higher eukaryotes [27].

Genomic regions of eukaryotes and prokaryotes with microsatellites are prone to genomic alterations particularly insertions and deletions [28]. In this work we observed such modifications when we compared the genomes of *A. fumigatus* and *N. fischeri* in regions with microsatellites. The motif length (tri-, tetra- or pentanucleotide) was not correlated with an increased presence in closely related species. Such genomic dynamics resulted in a distinct electrophoretic profile for each species following the application of microsatellite-based PCR multiplex at 55°C. The relevant pathogens of section *Fumigati*, such as *A. fumigatiaffinis, N. fischeri* and *N. udagawae*, were easily identified, however, testing this strategy in a broader range of species and isolates would better support identification of species within *Aspergillus* section *Fumigati*. This strategy has been successfully tested before in the identification of microsatellite transferability in close related species [29]. Furthermore, the genotyping strategies of less studied species of section *Fumigati* can now be better approached, as new microsatellite markers have now been proposed for *A. unilateralis* and *N. fischeri*.

Wide application of typing methodologies can give pertinent information regarding microbial epidemiology, chronic colonization for several patients and effectiveness of antibiotic treatments [11-14]. The initial question on the real specificity of the microsatellite markers selected for *A. fumigatus* genotyping was answered in the present work and it represents a genuine and required improvement for applicability of the methodology. We proved that the proposed panel with eight microsatellites [11] is highly appropriate for genotyping *A. fumigatus*. Besides genotyping, microsatellite-based multiplex PCR allows the identification of *A. fumigatus* and a slight modification of PCR conditions also allow identifying other pathogenic species within section *Fumigati*, particularly *A. fumigatiaffinisN. fischeri*, and *N. udagawae*. Sequence analysis of marker MC6b showed that *A. lentulus* and *A. viridinutans* were different from all the other tested species.

## Methods

### Fungal strains and culture conditions

A set of fungal isolates described as belonging to *Aspergillus* section *Fumigati* was obtained from Centraalbureau voor Schimmelcultures (CBS): the pathogenic moulds *Aspergillus fumigatiaffinis* (CBS 117186), *Aspergillus lentulus* (CBS 116880, 117180, 117182, and 117885), *Aspergillus viridinutans* (CBS 121595), *Neosartorya fischeri* (CBS 316.89), *Neosartorya hiratsukae* (CBS 124073), *Neosartorya pseudofischeri* (CBS 208.92 and 110899), and *Neosartorya udagawae* (CBS 114217), and two non-pathogenic moulds *Aspergillus novofumigatus* (CBS 117519) and *Aspergillus unilateralis* (CBS 126.56). The reference strain *A. fumigatus* ATCC 46645 was also included in the present work, as well as ten different strains of *A. fumigatus* from our collection. Monospore isolates from all the fungal strains were cultured on Sabouraud dextrose agar for 5 days at 30°C. A sodium hydroxide based method was used to extract DNA from fungal conidia (protocol at http://www.aspergillus.org.uk/indexhome.htm?secure/laboratory_protocols). Fungal DNA was suspended in 50 μl of sterile water and frozen at -20°C. Control of the DNA quality was carried out by amplifying and sequencing the β-tubulin region in all tested fungi, using previously selected primers [10].

### Microsatellite-based PCR multiplex

Microsatellite PCR multiplex was performed according to previously selected primers that allowed the identification of the microsatellites based on tri-, tetra-, and pentanucleotide motifs located in different chromosomes [11]. The PCRs were performed in 5 μL final volume, with 1 μL of genomic DNA (1–5 ng/μL), 2.5 μL of 2 × Qiagen multiplex PCR master mixes (Qiagen, Hilden, Germany) and 0.5 μL of a mix of eight primer pairs, at 2 μM concentration. After a 95°C preincubation step of 15 min, PCRs were performed for a total of 30 cycles, using the following conditions: denaturation at 94°C for 30 s, annealing at 60°C for 90 s and extension at 72°C for 1 min; with a final extension step of 10 min at 72°C. The internal size standard GeneScan 500 LIZ (Applied Biosystems, Foster City, CA, USA) (0.5 μL) and HiDiformamide (Applied Biosystems) (12 μL) were added to the PCR-amplified products and run in an ABI PRISM 3100 genetic analyser 16-capillary electrophoresis system (Applied Biosystems). Fragment size was performed automatically using Genemapper software 4.0 (Applied Biosystems).

### DNA sequencing conditions

PCR-generated fragments were purified with ExoSAP-IT (USB Corporation, Cleveland, Ohio, USA) and the reactions were conducted employing an ABI Big Dye terminator cycle sequencing kit (Applied Biosystems) under the following conditions: after a 95°C pre-incubation step of 15 min and DNA denaturation at 96°C for 15 s; 35 PCR cycles were performed with primer annealing at 50°C for 9 s, an extension at 60°C for 2 min; followed by a final extension at 60°C for 10 min. A volume of 8 μL of HiDiformamide were added to the sequencing products and run in an ABI PRISM 3100 Genetic Analyser 16-capillary electrophoresis system. The results were analyzed using the Sequencing 5.2 analysis software (Applied Biosystems).

### Data analysis

Complete genome sequences of *A. fumigatus* AF293 and *N. fischeri* NRRL 181 available at Ensembl (http://www.ensembl.org/index.html) were downloaded and the group of eight STRs located in those genomes employing the Geneious software v4.7 (Biomatters Ltd, Auckland, New Zealand) and BioEdit sequence alignment editor (available at http://www.ctu.edu.vn/~dvxe/Bioinformatic/Software/BioEdit.htm).

## Competing interest

No conflicts of interest. The authors have no financial relationship with the organizations that sponsored the research.

## Authors’ contributions

RA carried out the experimental studies. RA, AA, and LG conceived the study, participated in its design and coordination and drafted the manuscript. All authors read and approved the final manuscript.

## Supplementary Material

Additional file 1 Supplementary Table A1. Click here for file

Additional file 2 Figure A1. Click here for file

## References

[B1] Balajee SA Gribskov J Brandt M Ito J Fothergill A Marr KA Mistaken identity:Neosartoryapseudofischeriand its anamorph masquerading asAspergillus fumigatus J ClinMicrobiol 2005 43 5996 5999 10.1128/JCM.43.12.5996-5999.2005 PMC1317194 16333088

[B2] Balajee SA Gribskov JL Hanley E Nickle D Marr KA Aspergillus lentulussp. nov., a new sibling species ofA.fumigatus Eukaryot Cell 2005 4 625 632 10.1128/EC.4.3.625-632.2005 15755924 PMC1087803

[B3] Samson RA Hong S Peterson SW Frisvad JC Varga J Polyphasic taxonomy ofAspergillussectionFumigatiand its teleomorphNeosartorya Stud Mycol 2007 59 147 203 10.3114/sim.2007.59.14 18490953 PMC2275200

[B4] Balajee SA Nickle D Varga J Marr KA Molecular studies reveal frequent misidentification ofAspergillus fumigatusby morphotyping Eukaryot Cell 2006 5 1705 1712 10.1128/EC.00162-06 17030996 PMC1595351

[B5] Hong SB Shin HD Hong J Frisvad JC Nielsen PV Varga J Samson RA New taxa ofNeosartoryaandAspergillusinAspergillussectionFumigati Antonie Van Leeuwenhoek 2008 93 87 98 10.1007/s10482-007-9183-1 17610141 PMC2140094

[B6] Yaguchi T Horie Y Tanaka R Matsuzawa T Ito J Nishimura K Molecular phylogenetics of multiple genes onAspergillussectionFumigatiisolated from clinical specimens in Japan Jap J Med Mycol 2007 48 37 46 10.3314/jjmm.48.37 17287721

[B7] Brandt ME Padhye AA Mayer LW Holloway BP Utility of random amplified polymorphic DNA PCR and TaqMan automated detection in molecular identification ofAspergillus fumigatus J ClinMicrobiol 1998 36 2057 2062 10.1128/jcm.36.7.2057-2062.1998 PMC104978 9650962

[B8] Staab JF Balajee SA Marr KA AspergillusSectionFumigatityping by PCR-restriction fragment polymorphism J ClinMicrobiol 2009 47 2079 2083 10.1128/JCM.00551-09 PMC2708504 19403766

[B9] Etienne KA Gade L Lockhart SR Diekema DJ Messer SA Pfaller MA Balajee SA Screening of a large globalAspergillus fumigatusspecies complex collection by using a species-specific microsphere-based Luminex assay J Clin Microbiol 2009 47 4171 4172 10.1128/JCM.01415-09 19794043 PMC2786642

[B10] Serrano R Gusmão L Amorim A Araujo R Rapid identification ofAspergillus fumigatuswithin sectionFumigati BMC Microbiol 2011 11 82 10.1186/1471-2180-11-82 21510879 PMC3102036

[B11] Araujo R Pina-Vaz C Rodrigues AG Amorim A Gusmão L Simple and highly discriminatory microsatellite-based multiplex PCR forAspergillus fumigatusstrain typing Clin Microbiol Infect 2009 15 260 266 10.1111/j.1469-0691.2008.02661.x 19196262

[B12] Amorim A Guedes-Vaz L Araujo R Susceptibility to five antifungals ofAspergillus fumigatusstrains isolated from chronically colonised cystic fibrosis patients receiving azole therapy Int J Antimicrob Agents 2010 35 396 399 10.1016/j.ijantimicag.2009.12.007 20138740

[B13] Balajee SA de Valk HA Lasker BA Meis JF Klaassen CH Utility of a microsatellite assay for identifying clonally related outbreak isolates ofAspergillus fumigatus J Microbiol Methods 2008 73 252 256 10.1016/j.mimet.2008.02.011 18375005

[B14] Vanhee LM Symoens F Bouchara JP Nelis HJ Coenye T High-resolution genotyping ofAspergillus fumigatusisolates recovered from chronically colonised patients with cystic fibrosis Eur J Clin Microbiol Infect Dis 2008 27 1005 1007 10.1007/s10096-008-0527-1 18458971

[B15] Hanafy A Kaocharoen S Jover-Botella A Katsu M Iida S Kogure T Gonoi T Mikami Y Meyer W: Multilocus microsatellite typing for Cryptococcus neoformans var. grubii. Med Mycol 2008 46 685 696 10.1080/13693780802027062 18608927

[B16] Hennequin C Thierry A Richard GF Lecointre G Nguyen HV Gaillardin C Dujon B Microsatellite typing as a new tool for identification ofSaccharomyces cerevisiaestrains J Clin Microbiol 2001 39 551 559 10.1128/JCM.39.2.551-559.2001 11158105 PMC87774

[B17] Matute DR Sepulveda VE Quesada LM Goldman GH Taylor JW Restrepo A McEwen JG Microsatellite analysis of three phylogenetic species ofParacoccidioidesbrasiliensis J ClinMicrobiol 2006 44 2153 2157 10.1128/JCM.02540-05 PMC1489427 16757613

[B18] Sabino R Sampaio P Rosado L Stevens DA Clemons KV Pais C New polymorphic microsatellite markers able to distinguish amongCandida parapsilosissensu stricto isolates J Clin Microbiol 2010 48 1677 1682 10.1128/JCM.02151-09 20220157 PMC2863883

[B19] Kuhls K Keilonat L Ochsenreither S Schaar M Schweynoch C Presber W Schönian G Multilocus microsatellite typing (MLMT) reveals genetically isolated populations between and within the main endemic regions of visceral leishmaniasis Microb Infect 2007 9 334 343 10.1016/j.micinf.2006.12.009 17307010

[B20] Fedorova ND Khaldi N Joardar VS Maiti R Amedeo P Anderson MJ Crabtree J Silva JC Badger JH Albarraq A Angiuoli S Bussey H Bowyer P Cotty PJ Dyer PS Egan A Galens K Fraser-Liggett CM Haas BJ Inman JM Kent R Lemieux S Malavazi I Orvis J Roemer T Ronning CM Sundaram JP Sutton G Turner G Venter JC White OR Whitty BR Youngman P Wolfe KH Goldman GH Wortman JR Jiang B Denning DW Nierman WC Genomic islands in the pathogenic filamentous fungusAspergillus fumigatus PLoS Genet 2008 4 e1000046 10.1371/journal.pgen.1000046 18404212 PMC2289846

[B21] Sugui JA Vinh DC Nardone G Shea YR Chang YC Zelazny AM Marr KA Holland SM Kwon-Chung KJ Neosartorya udagawae(Aspergillus udagawae), an emerging agent of aspergillosis: how different is it fromAspergillus fumigatus? J Clin Microbiol 2010 48 220 228 10.1128/JCM.01556-09 19889894 PMC2812273

[B22] Padhye AA Godfrey JH Chandler FW Peterson SW Osteomyelitis caused byNeosartoryapseudofischeri J ClinMicrobiol 1994 32 2832 2836 10.1128/jcm.32.11.2832-2836.1994 PMC264168 7852580

[B23] Guarro J Kallas EG Godoy P Karenina A Gené J Stchigel A Colombo AL Cerebral aspergillosis caused byNeosartorya hiratsukae, Brazil Emerg Infect Dis 2002 8 989 991 10.3201/eid0809.020073 12194781 PMC2732550

[B24] Alcazar-Fuoli L Mellado E Alastruey-Izquierdo A Cuenca-Estrella M Rodriguez-Tudela JL AspergillussectionFumigati: antifungal susceptibility patterns and sequence-based identification Antimicrob Agents Chemother 2008 52 1244 1251 10.1128/AAC.00942-07 18212093 PMC2292508

[B25] Barada G Basma R Khalaf RA Microsatellite DNA identification and genotyping ofCandida albicansfrom Lebanese clinical isolates Mycopathologia 2008 165 115 125 10.1007/s11046-008-9089-0 18266073

[B26] Cristancho M Escobar C Transferability of SSR markers from related Uredinales species to the coffee rustHemileiavastatrix Genet Mol Res 2008 7 1186 1192 10.4238/vol7-4gmr493 19048497

[B27] Baird RE Wadl PA Allen T McNeill D Wang X Moulton JK Rinehart TA Abbas HK Shier T Trigiano RN Variability of United States isolates ofMacrophominaphaseolinabased on simple sequence repeats and cross genus transferability to related genera within botryosphaeriaceae Mycopathologia 2010 170 169 180 10.1007/s11046-010-9308-3 20352493

[B28] McDonald MJ Wang WC Huang HD Leu JY Clusters of nucleotide substitutions and insertion/deletion mutations are associated with repeat sequences PLoS Biol 2011 9 e1000622 10.1371/journal.pbio.1000622 21697975 PMC3114760

[B29] Dutech C Enjalbert J Fournier E Delmotte F Barrès B Carlier J Tharreau D Giraud T Challenges of microsatellite isolation in fungi Fungal Genet Biol 2007 44 933 949 10.1016/j.fgb.2007.05.003 17659989

