# Supplementary material for: Diversity and specificity of microsatellites within Aspergillus section Fumigati
Source: BMC Microbiol. 2012 Jul 28;12:154. doi: 10.1186/1471-2180-12-154 (PMC3438126; doi:10.1186/1471-2180-12-154)
Supplement: Additional file 1 — Supplementary Table A1. [file 1471-2180-12-154-S1.doc]

**Supplementary Table A1**. Sequence analysis of the microsatellites amplified at 50 ºC in species belonging to section *Fumigati* compared with the reference sequence of *Aspergillus fumigatus* AF293 and *Neosartorya fischeri* NRRL 181 (microsatellites are marked blue). Sequences are given in orientation 5’ to 3’.

| MC8 | *A. fumigatus* AF293  *Neosartorya udagawae* CBS 114217 | TGCTTC (CATA)8 CATCCATACATC  TGCAAT CATA C CATGAGCACATC |
| --- | --- | --- |
| MC5 | *A. fumigatus* AF293  *Aspergillus fumigatiaffinis* CBS 117186  *Aspergillus novofumigatus* CBS 117519  *Neosartorya fischeri* NRRL181  *Neosartorya fischeri* CBS 316.89 | CCTTCAGCTTTGCTATTTA (TTTAT)17(TTTA)3 TTTCTTTATTATTATTATTTTTATTTCCG  CCTTCAGCTTTGTTA---- (TTTTTTA)2 ----------------TTTTTTATTTCCG  CCTTCAGCTTTGTTA---- (TTTTATTTTA)2 ---CTTT----------TTTTTATTTCCG  CCTTCAGCTTTGTTATTTT -----------------TTTTTATTTCCG  CCTTCAGCTTTGTTATTTT --------------TTTTTTTAATTTCCG |
| MC6a | *A. fumigatus* AF293  *Neosartorya fischeri* NRRL 181  *Neosartorya fischeri* CBS 316.89 | TGTTTCAATAAG (AAG)19 GCAACAGAATAACAC  TGTTTCAAATGT (AAG)4 GCAACAGAATAAGAC  TGTTTCAAATGT (AAG)4 GCAACAGAATAAGAC |
| MC6b | *A. fumigatus* AF293  *Aspergillus fumigatiaffinis* CBS 117519  *Aspergillus lentulus* CBS 116880  *Aspergillus unilateralis* CBS 126.56  *Aspergillus viridinutans* CBS 121595  *Neosartorya fischeri* NRRL 181  *Neosartorya fischeri* CBS 316.89  *Neosartorya pseudofischeri* CBS 208.92  *Neosartorya udagawae* CBS 114217 | CGCTACACG (GAAA)11 GAA-GATCA  CG-TA-A-G GAGAGAAA GAATGATCA  CG-TA-A-G (GAAA)2 GAATGACCA  C-C------ (GAAA)3 G----ATCA  CG-TA-A-G (GAAA)2 GAATGACCA  CG-TA-A-G (GAAA)2 GAATGATCA  CG-TA-A-G (GAAA)2 GAATGATCA  CG-TA-A-G GAGAGAAA GAATGATCA  CG-TA-A-G (GAAA)2 GAATGATCG |
