# Supplementary material for: Diversity and specificity of microsatellites within Aspergillus section Fumigati
Source: BMC Microbiol. 2012 Jul 28;12:154. doi: 10.1186/1471-2180-12-154 (PMC3438126; doi:10.1186/1471-2180-12-154)
Supplement: Additional file 2 — Figure A1. [file 1471-2180-12-154-S2.pdf]

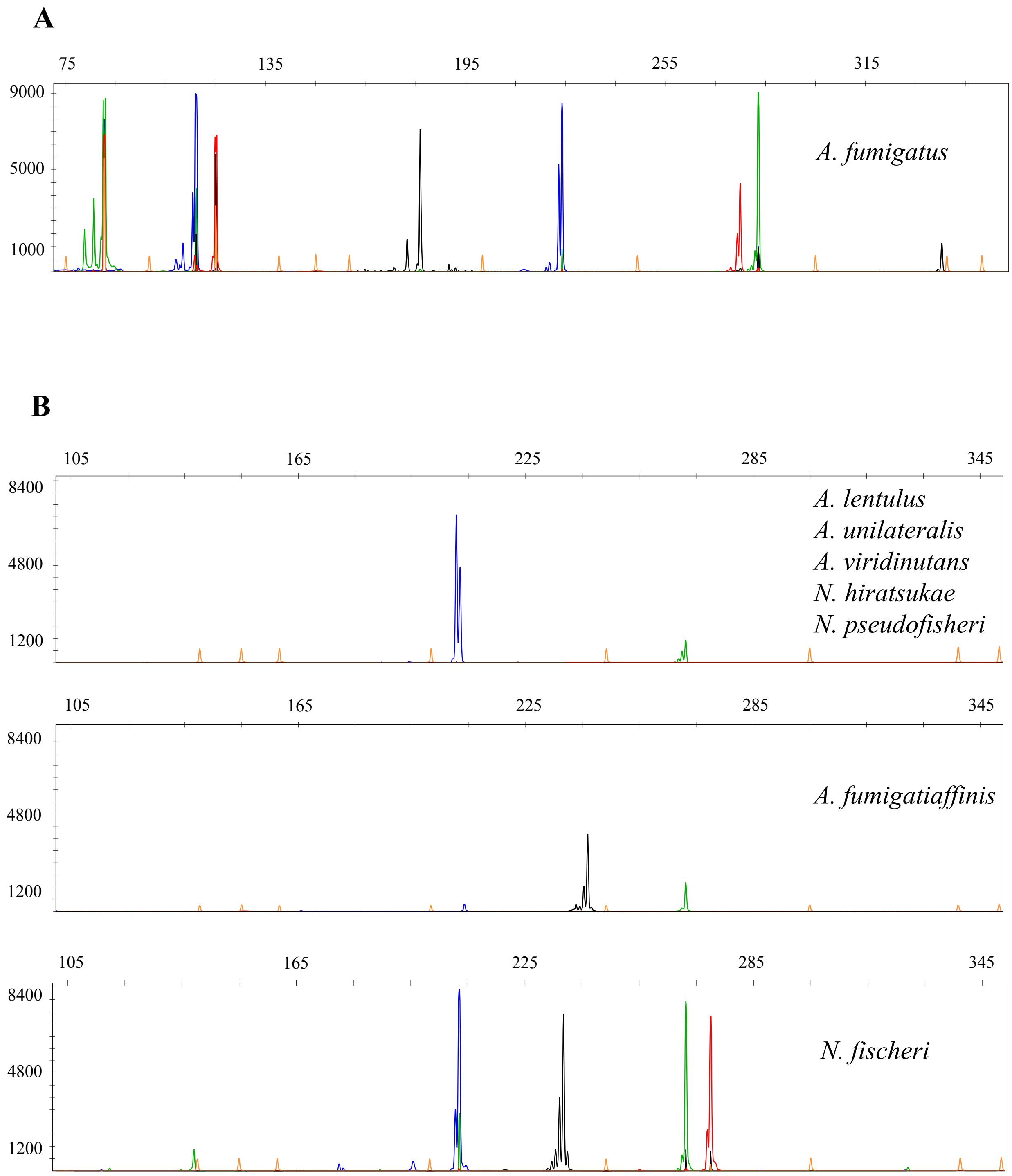


Figure A1. Microsatellite-based PCR multiplex: A) Profile of eight peaks typical for *A. fumigatus*; B) Profile obtained in species belonging to *Aspergillus* section *Fumigati*.
